# Noninvasive assessment of arterial compliance of human cerebral arteries with short inversion time arterial spin labeling

**DOI:** 10.1038/jcbfm.2014.219

**Published:** 2014-12-17

**Authors:** Esther AH Warnert, Kevin Murphy, Judith E Hall, Richard G Wise

**Affiliations:** 1 Cardiff University Brain Research and Imaging Centre (CUBRIC), School of Psychology, Cardiff University, Cardiff, UK; 2 Department of Anaesthetics and Intensive Care Medicine, School of Medicine, Cardiff University, Cardiff, UK

**Keywords:** arterial blood volume, arterial compliance, ASL, cerebral hemodynamics, middle cerebral artery, MRI

## Abstract

A noninvasive method of assessing cerebral arterial compliance (AC) is introduced in which arterial spin labeling (ASL) is used to measure changes in arterial blood volume (aBV) occurring within the cardiac cycle. Short inversion time pulsed ASL (PASL) was performed in healthy volunteers with inversion times ranging from 250 to 850 ms. A model of the arterial input function was used to obtain the cerebral aBV. Results indicate that aBV depends on the cardiac phase of the arteries in the imaging volume. Cerebral AC, estimated from aBV and brachial blood pressure measured noninvasively in systole and diastole, was assessed in the flow territories of the basal cerebral arteries originating from the circle of Willis: right and left middle cerebral arteries (RMCA and LMCA), right and left posterior cerebral arteries (RPCA and LPCA), and the anterior cerebral artery (ACA). Group average AC values calculated for the RMCA, LMCA, ACA, RPCA, and LPCA were 0.56%±0.2%, 0.50%±0.3%, 0.4%±0.2%, 1.1%±0.5%, and 1.1%±0.3% per mm Hg, respectively. The current experiment has shown the feasibility of measuring AC of cerebral arteries with short inversion time PASL.

## Introduction

The classic definition of arterial compliance (AC) by Spencer and Denison^1^ is a change in arterial blood volume (aBV) resulting from a given change in arterial blood pressure (BP). The compliance of arteries ensures that they are able to accommodate the pulsatile blood flow originating from the heart and average out these pulsations into continuous blood flow in the capillary bed of human tissue.^2^ When AC decreases, that is arteries become stiffer, they lose the ability to smooth the pulsatile blood flow and as a consequence downstream arterioles and capillaries are exposed to higher BP fluctuations.^3^ This increase in BP in the distal arterial bed in turn causes deterioration of vessel walls, which in the brain manifests as cerebral small-vessel disease (SVD).^3^ The prevalence of SVD increases with aging and has also been linked to cognitive decline in patients with dementia and Alzheimer's disease.^4^ Moreover, patients with diabetes mellitus^3^ and hypertension^5^ also have an increased risk at developing SVD. Although the mechanisms underlying development of cerebral SVD are still poorly understood, it is evident that arterial stiffening is highly linked to this pathologic assessment.^6^ Measuring local AC in the brain may therefore give valuable insight in the development and treatment of cerebral SVD.

Compliance of cerebral arteries has long been ignored in cerebrovascular research because of the difficulty of obtaining *in vivo* measurements of mechanical wall properties and the fact that *ex vivo* deformation testing has shown that intracerebral arteries are relatively stiff compared with extracranial arteries.^7, 8^ However, from a physiologic standpoint it is important that cerebral arteries are also compliant and act as a flow buffer to enable steady flow through the cerebral capillary bed and to protect this distal microvasculature from BP fluctuations.^9^ This is likely the reason for the fact that recent advances in modeling cerebral pressure–flow relationships have shown that including an AC component results in more physiologic flow profiles than using rigid arterial wall models.^8, 9, 10, 11^ In addition, biaxial deformation testing has shown that circumferential deformation in fresh human cerebral arteries occurs with much less resistance than axial deformation,^12^ highlighting that cerebral arteries are able to show compliant behavior.

Currently, ultrasound echotracking of arterial walls to obtain maximum (systolic) and minimum (diastolic) diameters with simultaneous measurement of local pulse pressure (the difference between systolic and diastolic BP) is one of the standard methods to assess local AC,^13, 14^ whether it be in peripheral (e.g., radial^15^) or more central (e.g., carotid^16^) arteries. However, echotracking of cerebral arteries is difficult because the skull reflects most of the ultrasound waves.^17^ One ultrasound method that is currently used to assess cerebral AC is transcranial Doppler ultrasound (TCD),^18, 19, 20^ which detects blood flow velocities from accessible cerebral arteries (mainly the middle cerebral artery (MCA)). One drawback of TCD is that it cannot measure arterial geometry, such as volume or diameter, directly and therefore either a mathematical model is used to transform blood flow into volume, which assumes that there is no change in arterial diameter,^18, 19^ or arterial stiffness is assessed with the augmentation index, a parameter based on the wave form of the blood flow velocity.^20^ Another drawback of TCD, in particular for the MCA, is that thickness of the temporal bone renders it impossible to measure MCA flow velocities in ~20% of the population.^21^

We propose a method that enables measurement of cerebral aBV based on short inversion time (TI) pulsed arterial spin labeling (PASL).^22^ Pulsed arterial spin labeling is a method in which arterial blood is magnetically labeled before it reaches the volume of interest and is usually applied to measure tissue perfusion, which is the delivery of blood to the capillary bed of the brain. Images containing perfusion-weighted signal are calculated by subtracting a tag image from a control image. The latter acquired without any labeling of arterial blood, but before acquisition of the tag image blood is labeled when it flows through the major brain-feeding arteries (i.e., internal carotid and vertebral arteries) and image acquisition takes place after leaving time (>1 second) for the labeled blood to pass into the cerebral capillary bed. However, before arriving at the capillary bed the labeled blood first has to pass through the macrovasculature of the brain. Imaging at short TIs (<1 second) therefore results in ASL images in which the major arteries, such as the MCAs, posterior and anterior cerebral arteries (PCAs and ACA), are clearly visible.

The aim of the study is to show the feasibility of noninvasive measurement of AC by exploiting the signal arising from the cerebral arteries at short TI PASL.

### Modeling Arterial Blood Volume

**Figure 1:**
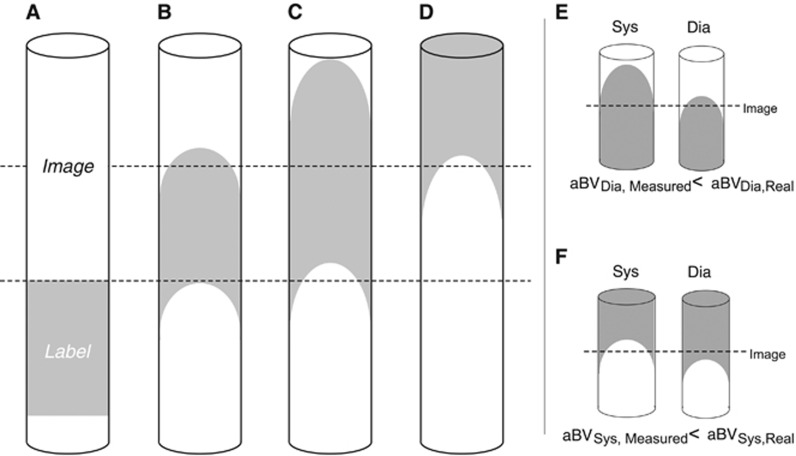
(**A**–**D**) Schematic of the labeled bolus in pulsed arterial spin labeling evolving over time. To measure arterial blood volume (aBV) with a single inversion time it is important that all the arterial blood in the image is labeled, as is the case in **C**. Owing to dispersion of the label there will be times when not all the arterial blood in the image is labeled, as is the case in **B** and **D**. Assessing aBV in systole (sys) and diastole (dia) with a single inversion time can be prone to error. When imaged too early, the diastolic blood volume is underestimated (**E**). When imaged too late the systolic blood volume is underestimated (**F**).

In measuring aBV with PASL it is important to account for changes in the kinetics of the labeled bolus because of the cardiac cycle, such as differences in bolus arrival time.^23, 24^ In addition, as the label progresses through the vasculature dispersion takes place, which means that the leading and trailing edges of the bolus are not well defined (Figures 1A and 1D). If a single TI is used to assess blood volume, the cardiac phase–related changes in label kinetics may cause underestimation of aBV, as illustrated by Figures 1E and 1F, which in turn will confound calculation of AC. To account for changes in arrival time and dispersion, we therefore model the aBV based on multi-TI PASL data.

Here we model the arterial signal by extending the macrovascular compartment described previously by Chappell *et al*,^22^ which assumes plug flow in large arteries, with a Gaussian kernel to account for dispersion as has been introduced previously by Wu *et al*:^23^


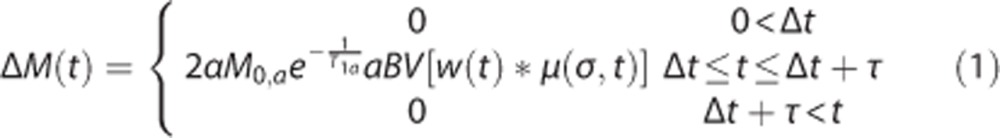


With ΔM(*t*) the difference signal arising from subtracting tag and control PASL images, aBV is the arterial blood volume in fraction of the voxel volume (%_v_), Δ*t* is the bolus arrival time (in ms), *τ* is the bolus duration (in ms), *α*=1 (label efficiency), and *T*_1,a_=1664 ms (*T*_1_ arterial blood at 3T).^22^ M_0,a_ can be determined based on the equilibrium magnetisation of cerebrospinal fluid (M_0,CSF_) according to methods previously described by Wong *et al.*^25^ Furthermore, *w*(*t*) is a square weighting function with the same width as the label duration (*τ*):


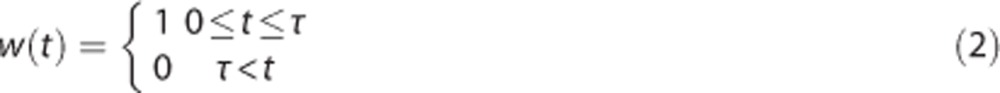


Which is convolved with a Gaussian kernel centered on Δ*t* and with width *σ* to model dispersion:


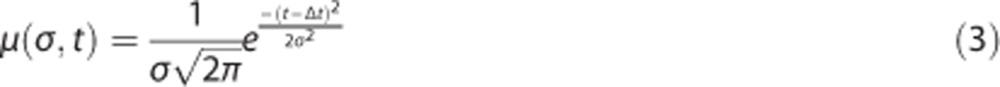


It is important to note that, although PASL is most commonly performed to assess tissue perfusion, here the interest lies solely in measuring the PASL signal of the macrovasculature. For this purpose, only short TI PASL with TIs <1 second are used and regions of interest (ROIs) are drawn that only contain voxels with a relatively large arterial compartment (see Materials and Methods section for more details). The short TIs ensure that the signal coming from the brain parenchyma is small in comparison to the signal coming from large arteries. Even though arrival times of the label into the microvasculature (Δ*t*_tiss_) have been reported to be as short as 700 ms for proximal brain regions (i.e., 40 mm from distal end of the labeling region^26^), the maximum tissue signal is still reported to occur at time points well over 1 second.^26, 27, 28^ In addition, the ROIs used here have a large macrovascular compartment, which ensures that the maximum macrovascular signal is in fact 6 to 10 times larger than the maximum tissue signal.^26^

**Figure 2:**
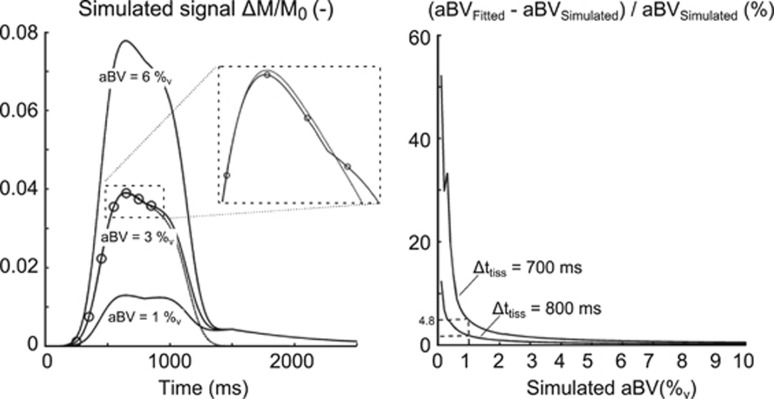
Left: Simulated ΔM/M_0_ signal from a voxel containing a macrovascular and microvascular compartment. The microvascular compartment was simulated with previously described methods,^22^ the cerebral blood flow was set to be 60 mL/100 g/min and the tissue arrival time (Δ*t*_tiss_) at 800 ms. Arrival time of the labeled bolus at the microvasculature was set to be 450 ms, and arterial blood volume (aBV) ranged from 0.1% to 10%. The resulting two-compartment model is plotted (solid lines) for aBV=1%, 3%, and 6%. Seven time points <1 second were then used (open circles) to fit Equation 1 to estimate aBV. An example of this fit is plotted (dotted line) for aBV=3%_v_. The inset shows how the fitting aBV with only the short inversion time slightly overestimates the true aBV. Right: The differences of the fitted aBV, based on the seven short inversion times, and the simulated aBV is plotted versus the simulated aBV (%_v_). The difference is plotted as a percentage change: 100% × (aBV_fitted_−aBV_simulated_)/aBV_simulated_. This plot shows that for Δ*t*_tiss_=700 ms and aBV_simulated_>1%, using only the short inversion times leads to overestimation of aBV of <5%. This overestimation becomes smaller with increasing aBV, and increasing Δ*t*_tiss_.

To confirm the minimal interference of signal coming from the microvasculature at short TIs, the signal from a voxel containing both macrovascular and microvascular compartments was simulated up to 2.5 seconds after PASL labeling by adding a tissue signal model described previously^22^ to Equation 1. The aBV was set to range between 0.1%_v_ and 10%_v_, the tissue perfusion was set at 60 mL/100 g/min and Δ*t*_tiss_ was 350 ms later than the arterial arrival time, which is in line with previous ASL studies that have included flow crushing gradients to estimate the macrovascular and microvascular arrival times.^26^ For each of the simulated signal curves, Equation 1 was then fitted to the signal at seven TIs (all <1 second) to give an estimate of aBV. Results from this simulation study showed that when the underlying aBV was larger than 1%_v_ the aBV calculated based on early TIs only, overestimates the true value by <5% for Δ*t*_tiss_=700 ms (Figure 2). With either larger underlying aBV or larger Δ*t*_tiss_, the overestimation of aBV becomes smaller. In the current experiment, the PASL data are therefore analyzed without the microvascular compartment and an estimate of tissue perfusion is not given.

## Materials and methods

Imaging data were acquired on a 3 T whole-body MRI system (GE Excite HDx, Milwaukee, WI, USA) using an eight-channel receive-only head coil. Informed consent was obtained from all volunteers under ethical approval from the Cardiff University School of Psychology Ethics Committee and all experiments were performed in accordance with the guidelines stated in the Cardiff University Research Framework (version 4.0, 2010).

### Image Acquisition

Multiinversion time PICORE^29^ PASL acquisitions were performed on five healthy volunteers (age 25.4±1.5 years). Seven TIs were acquired in random order (250 to 850 ms, spacing 100 ms, separate scan series). Note that all TIs <1 second, as explained in the Introduction section. The label was applied 10 mm below the most proximal slice and had a width of 200 mm. A Quantitative Imaging of Perfusion with a Single Subtraction (QUIPSS) II^25^ cut-off of the label was applied at 700 ms for TIs >700 ms. Images were acquired with a spiral gradient echo sequence (echo time (TE)=2.7 ms, repetition time (TR)=1400 ms, 80 tag-control pairs per TI, 14 slices, slice gap 1 mm, slice delay 29 ms, voxel size 3 × 3 × 7 mm^3^). A fully relaxed (infinite TR) calibration image was obtained with the same acquisition parameters but without labeling, to obtain the equilibrium magnetisation of CSF (M_0,CSF_).

### Blood Pressures

The cardiac cycle was monitored by finger plethysmography. Brachial artery BP was measured noninvasively with an MRI-compatible BP cuff once for each ASL scan (OMRON, Tokyo, Japan). Per participant, the seven BP measurements were used to calculate average systolic and diastolic BP (BP_sys_ and BP_dia_).

### Image Analysis

Pulsed arterial spin labeling time series were motion corrected using *mcflirt* within the FMRIB Software Library v5.0 (FSL, http://fsl.fmrib.ox.ac.uk).^30^ Retrospective synchronization was then performed using in-house programs written in MATLAB R2012b (MathWorks, Natick, MA, USA). A time shift of 225 ms was applied to the finger plethysmograph trace to account for the delay between the cerebral and finger pulse. This time period was deduced from the delay time between the *R*-peak in the electrocardiogram and the left finger, and the time delay between *R*-peak in the electrocardiogram and the onset of the carotid pulse wave.^31^ Images were further analyzed on slice-by-slice basis, taking into account the slice time delay. The normalized cardiac phase (*ϕ*_c_) of each acquired slice was determined according the following:


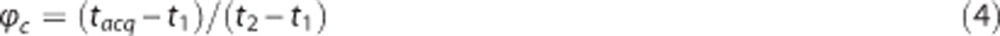


Where *t*_acq_ is the time of the slice acquisition, *t*_1_ is the time of the previous systolic peak in the shifted plethysmography trace, and *t*_2_ is the time of the following systolic peak in the plethysmography trace. Thus, the cardiac cycle is then advancing linearly from 0 to 1 during a single cardiac cycle and is then set to 0 for the next cardiac period. The cardiac cycle was then divided into eight bins, i.e., the first bin containing slices acquired with 0⩽*ϕ*_c_<1/8, the second bin containing slices acquired with 2/8⩽*ϕ*_c_<3/8 and so on. Slices acquired in ‘early diastole' corresponded to those with 1/8⩽*ϕ*_c_⩽2/8 and slices acquired in ‘early systole' were those with 6/8⩽*ϕ*_c_⩽7/8. Average tag and control images were calculated and subtracted to obtain maps of ΔM for each of the eight cardiac phases at each of the seven TIs.

The number of tag and control images in each cardiac phase was noted to analyze whether the synchronization led to any bias for a particular cardiac phase or TI.

### Calculation of Parameter Maps

Equation 1 was used to estimate aBV, Δ*t*, and *σ* on a voxel-wise basis for each of the eight cardiac phases separately (least-square fitting, *lsqcurvefit* in MATLAB (MathWorks)). The additional slice acquisition delay of 29 ms for each subsequent slice was taken into account in the fitting procedure. The bolus duration (*t*) was fixed at 700 ms by the QUIPSS II cut-off. Maps of aBV_Dia_ and aBV_Sys_ were used to calculate AC according to:


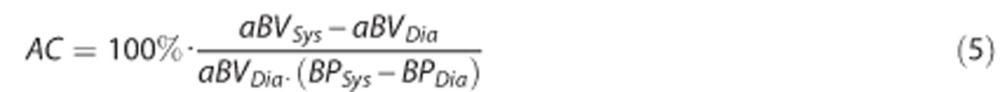


Note that here AC is normalized for the aBV in diastole and is therefore calculated as percentage change in aBV per mm Hg (%/mm Hg), which can also be referred to as arterial distensibility.^32^

### Regions of Interest

For each participant, an average ΔM image was obtained for the full time series of TI=750 ms (i.e., the average of 80 tag-control difference images). Regions of interest were determined based on data from TI=750 ms because on average this was the TI with the maximum intensity in the raw difference images (data not shown). A mask of the TI=750 ms ΔM image was created such that each slice only contained the 5% of voxels with the highest intensities (the 95th percentile intensity threshold was determined for each slice separately). Broad ROIs were manually drawn around the vascular territories within each slice. For instance, in the slice just above the circle of Willis these regions were drawn to encompass the flow territories of the right middle, left middle, right posterior, left posterior, and anterior cerebral arteries (RMCA, LMCA, RPCA, LPCA, and ACA). The ROIs of the flow territories were then calculated by multiplying each of the broad masks with the thresholded ΔM image. Note that there is only one ROI for the ACA, because the voxel size used for image acquisition did not allow for separation of the left and right ACA.

### Statistical Analysis

A three-way repeated measures analysis of variance (RM-ANOVA) was used to assess the number of tag and controls per cardiac phase after retrospective synchronization of image acquisition with the cardiac cycle. TI, ROI, and cardiac phase (systole versus diastole) were used as independent variables. To investigate differences in aBV, Δ*t*, and *σ* between flow territories and between cardiac phase a two-way RM-ANOVA was performed with ROI and cardiac phase (systole versus diastole) as independent variables. Note that only the diastolic and systolic parameter maps are used for the above RM-ANOVAs. To assess differences in AC between different flow territories a one-way RM-ANOVA was performed, with ROI as the independent variable.

## Results

All relevant subject information is summarized in Table 1. The average pulse pressure (BP_Sys_–BP_Dia_) was 52.7±9.5 mm Hg with an average heart rate of 65.4±10.0 beats per minute.

Retrospective resynchronization of image acquisition with the cardiac cycle did not lead to any bias toward a particular TI (RM-ANOVA, F(6,24)=0.804, *P*=0.557). On average, out of the 160 images acquired for a single TI there were 10 tag and 10 control images in diastole, and 10 tag and 11 control images in systole.

**Figure 3:**
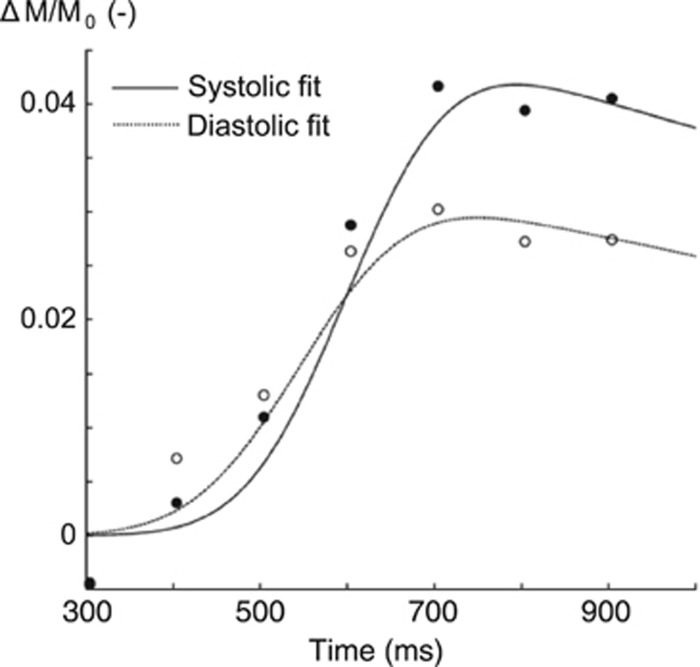
Example of model fit for a single voxel in the flow territory of the right middle cerebral arteries of one subject. Note that although signal intensities were calculated for eight different cardiac phases, only systolic (•) and diastolic (∘) data points are shown here. Data were corrected for slice time acquisition.

**Figure 4:**
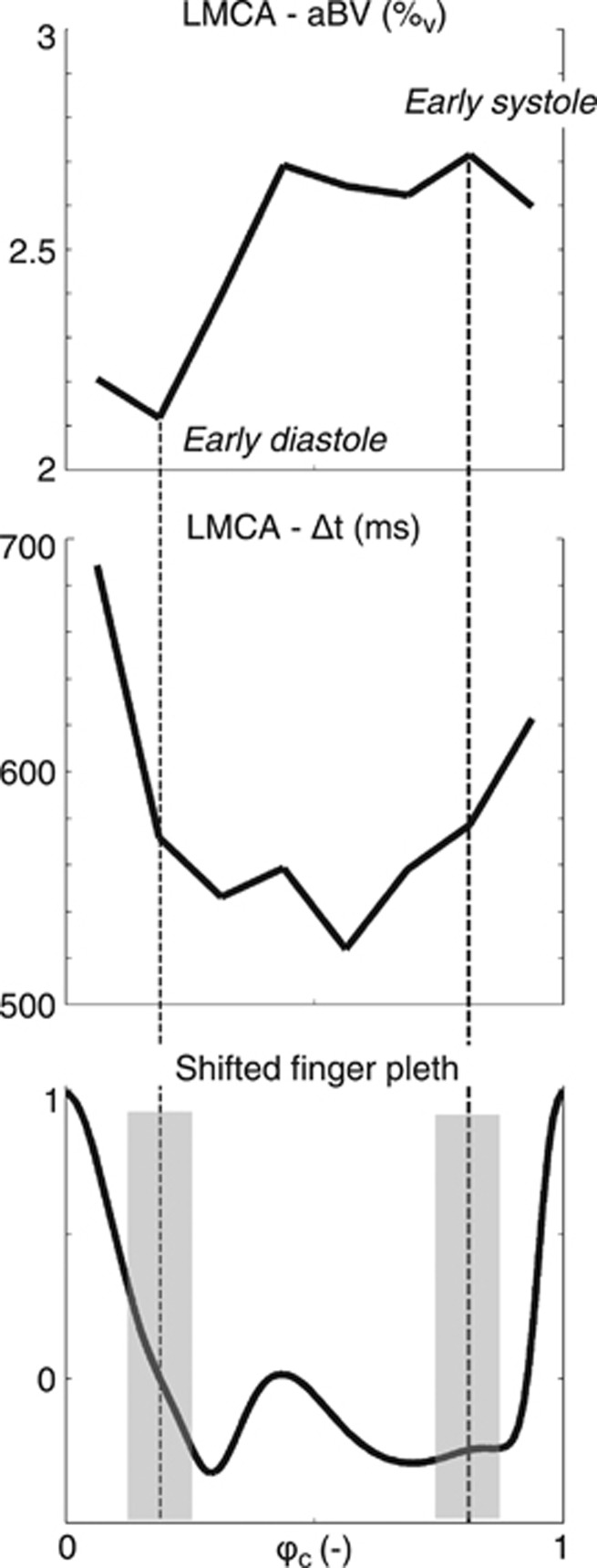
Example of fitted aBV (top) and Δ*t* (middle) calculated for each of the eight cardiac phases. The full trace of the cardiac cycle can be seen in the bottom image. This data is from the LMCA flow territory of subject V. Note how these images clearly show cardiac cycle–related variation in aBV and Δ*t*. Images acquired in early diastole had 0.125⩽*ϕ*_c_⩽0.25 and in early systole had 0.75⩽*ϕ*_c_⩽0.875 (gray bars in the bottom image). aBV, arterial blood volume; LMCA, left middle cerebral artery.

**Figure 5:**
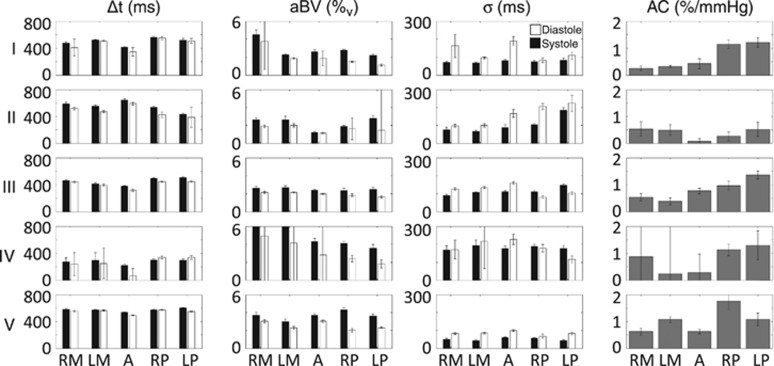
Regional median results for aBV, Δ*t*, *σ*, and AC for each individual subject (I–V) calculated for the slice just above the circle of Willis. Arterial blood volume, Δ*t*, and *σ* are given for systole (black) and diastole (white). Arterial compliance is calculated as percentage of increase in aBV in systole compared with diastole. Errorbars indicate 1 s.e.m. Repeated measures analysis of variance showed that aBV_Sys_>aBV_Dia_ for each ROI (pairwise comparisons, *P*<0.05, after a significant effect of the interaction between ROI and systole versus diastole was found, F(4,16)=4.2, *P*<0.03). A, anterior cerebral artery; aBV, arterial blood volume; AC, arterial compliance; LM, left middle cerebral artery; LP, left posterior cerebral artery; RM, right middle cerebral artery; ROI, region of interest; RP, right posterior cerebral artery.

Fitting the model on a voxel-by-voxel basis leads to individual parameter maps for aBV, Δ*t*, and *σ* calculated for eight different cardiac phases. An example of fitting the model to data from a single voxel in early systole (6/8⩽*ϕ*_c_<7/8) and early diastole (1/8⩽*ϕ*_c_<2/8) can be seen in Figure 3. Maps of the root mean square error of the model fitting can be found in Supplementary Figure 1. Regional aBV and Δ*t* showed consistent variation along the cardiac cycle in all participants (an example can be seen in Figure 4). Median values were calculated for the aforementioned five flow territories just above the circle of Willis in systole and diastole (Figure 5). Repeated measures ANOVA showed that aBV_Sys_ was significantly higher than aBV_Dia_ for each of the five ROIs (pairwise comparisons, *P*<0.05, after RM-ANOVA showed significant effect of cardiac phase on aBV F(1,4)=16.5, *P*<0.02). Also, on average Δ*t*_Sys_ was 41 ms longer than Δ*t*_Dia_ (RM-ANOVA significant effect of systole versus diastole, F(1,4)=22.9, *P*<0.01). In addition, *σ*_Dia_ was on average 30 ms larger than *σ*_Sys_ for the LMCA and ACA, although this was not significant (RM-ANOVA, effect of cardiac phase on *σ* F(1,4)=7.37, *P*=0.0533).

Individual values for AC for the slice just above the circle of Willis can be seen in Figure 5. Averaged over five subjects AC values calculated for the RMCA, LMCA, ACA, RPCA, and LPCA were 0.57%±0.20%, 0.50%±0.30%, 0.43%±0.24%, 1.1%±0.48%, and 1.1%±0.30% per mm Hg, respectively. Note that these values for AC indicate that we have measured aBV changes within the cardiac cycle from 25% to 50% (multiplying AC by the pulse pressure).

**Figure 6:**
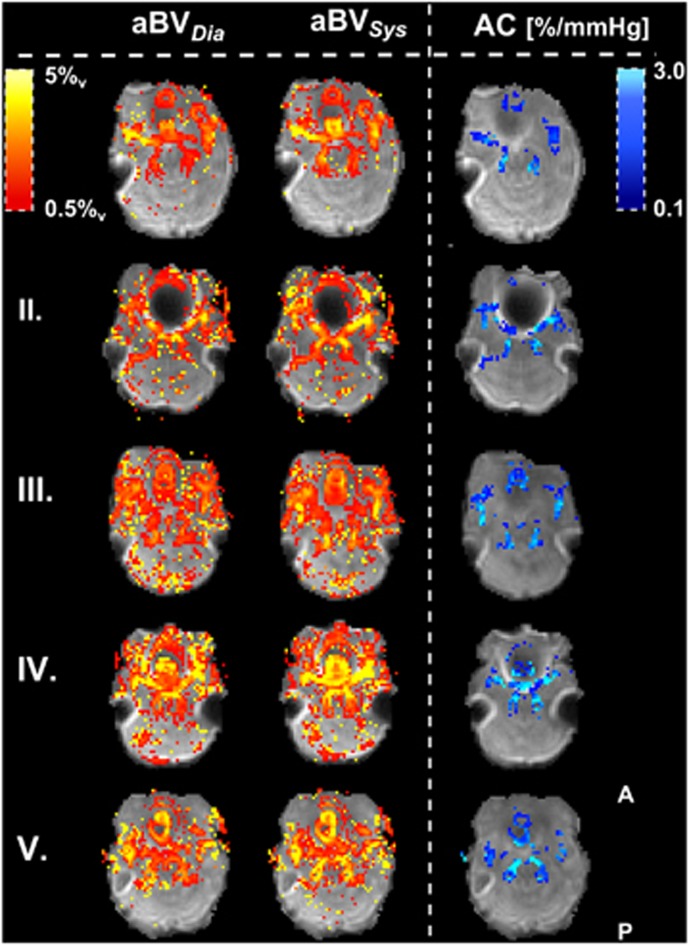
Maps of aBV (%_v_) just above the circle of Willis. aBV_Dia_ and aBV_Sys_ are plotted for each individual (top row shows subject I). The calculated AC maps are plotted in the right column. Note how AC here is normalized for aBV_Dia_ and is therefore showing percentage increases in aBV between systole and diastole per mm Hg increase in blood pressure. The AC maps are masked, showing only the voxels used to calculate flow territory averages. aBV, arterial blood volume; AC, arterial compliance.

Examples of the aBV maps in diastole and systole and the resulting maps of AC containing the slice just superior to the circle of Willis can be seen in Figure 6.

## Discussion

The current study has shown the feasibility of noninvasive assessment of AC of major cerebral arteries by retrospective synchronization of short TI ASL images with the cardiac cycle. In addition to finding significant differences between systolic and diastolic aBV, we have also found differences in dispersion and arrival time, which can be attributed to the pulsatility of the cardiac cycle.

### Pulsatility of the Cardiac Cycle

Although it has been shown that pulsatility arising from the cardiac cycle causes modulation of PASL signal,^23, 33^ to our knowledge this is the first study actually exploiting this modulation of PASL signal to assess cerebrovascular AC. In particular, here we robustly show that aBV changes in line with the cardiac cycle (Figure 4), which is as expected based on elastic properties of the cerebral arteries^34^ as well as on AC measured in arteries outside the brain.^32^

One important aspect of assessing cardiac pulsatility of the PASL signal in the *imaging* volume is accounting for the effect that the cardiac phase of the *labeling* slab has on PASL signal. That is, it is important to ensure that the changes in aBV we find in the *imaging* volumes are truly caused by changes in arterial volumes and not by differences caused by labeling at different cardiac phases. For instance, previous work concerning PICORE PASL with a QUIPSS II cut-off at 700 ms has shown that between applying the label in systole or diastole there can be differences in perfusion-weighted signal of up to 16%.^23^ To investigate the effect of the cardiac phase on the label, the PASL time series acquired here were also retrospectively synchronized to the cardiac cycle based on the cardiac phase of the label. Effectively, an image volume for a single cardiac phase now exists of slices that result from labeling at a fixed time within the cardiac cycle, meaning that each *imaging* slice will be acquired in a later cardiac phase because of the presence of the slice delay. Maps for aBV and Δ*t* were calculated by fitting the model to these images the same way as described in the Materials and Methods section. The main finding from this analysis is that the cardiac phases with maximum and minimum aBV (*ϕ*_aBV,max_ and *ϕ*_aBV,min_) resulting from synchronization according to cardiac phase of the label showed a dependency on slice location, shifting along the cardiac cycle for more distal slices (Illustrated by Supplementary Figure 2).

The fact that *ϕ*_aBV,max_ and *ϕ*_aBV,min_ shift for more distal slices when the label is applied in the same phase for each of the slice acquisitions shows that the aBV within the slice follows the cardiac phase of the slice itself, rather than being determined by the cardiac phase of the label location at the time of labeling. An important note here is that, when images are sorted according to the cardiac phase of the label and 14 slices are acquired with a slice delay of 29 ms, there is a cardiac phase difference (Δ*ϕ*_c_) of more than 0.4 (406/1,000 ms) between the most proximal and most distal slice acquired for the average heart rate of 60 beats per minute (*R*–*R* interval of 1000 ms). The fact that this phase shift is measurable with the aBV resulting from data that are synchronized based on when the label is acquired shows that the effect of the cardiac phase of the label on assessing aBV in the *imaging* volume is less important than the cardiac phase of the slice itself. Moreover, if there were a significant effect of the cardiac phase of the label, a similar dependency of *ϕ*_aBV,max_ and *ϕ*_aBV,min_ on slice location would be present in the dataset that is sorted based on the cardiac phase of the *imaging* slice. The fact that systolic and diastolic phase are the same in almost all slices in this study (i.e., early systole: 6/8⩽*ϕ*_c_<7/8 and early diastole 1/8⩽*ϕ*_c_<2/8), suggests that the effect of the cardiac phase of the label is indeed minimal (illustrated by Supplementary Figure 2).

In short, the findings from the analysis in which the PASL time series were sorted according to the cardiac phase of the *label* show that even if there is an effect of the cardiac phase of the label on measured aBV, this effect is small relative to the change in arterial volume caused by the cardiac cycle in the *imaging* volume and is therefore considered negligible in this study.

### Previously Reported Arterial Compliance of Cerebral Arteries

Owing to the difficulty of *in vivo* measurement of local cerebral AC, reference values for healthy cerebral arteries are difficult to find. Interestingly, Giller *et al*^35^ performed measurements of outer arterial diameters during craniotomies of 10 patients with varying pathologies and from their data it can be derived that they measured changes in volume related to changes in BP in exposed MCAs (M2 segment) and ACAs of 0.6% and 0.9% per mm Hg, respectively. Our volume-related changes for the MCA (0.54%/mm Hg, averaged over right and left) compare well with derived volume changes as reported by Giller *et al*,^35^ while it appears that here we underestimate the compliance in the ACA (0.43%/mm Hg). It is important to note, however, that the circumstances in which Giller *et al*^35^ measured changes in arterial diameters may not be reflective of the true *in vivo* AC as several factors during craniotomy may affect compliance and pulsatile behavior of exposed arteries, such as a change in transmural pressure of the artery owing to its exposure, the anesthesia used, and the pathologic assessment present. However, it remains of interest that, despite the obvious differences in methods of measuring AC, Giller *et al* do measure larger changes in diameter of smaller arteries, which is also suggested by our finding of significantly larger AC in the PCA segments within the circle of Willis.

A direct comparison between AC reported here and AC methods derived from TCD should be interpreted with caution. As mentioned in the introduction, TCD (of the MCA) is restricted to the measurement of blood flow velocity and to estimate compliance, assumptions on vessel diameter have to be made. For instance, in the method described by Kim *et al*,^19^ the assumption is made that there is no change in MCA diameter, neither over the cardiac cycle nor when intracranial pressure increases, and the MCA velocity profile is then used to model arterial inflow and venous outflow of the arterial bed distal to the location of insonation. The compliance measure that is calculated then does not reflect compliance of the MCA, but of the arterial bed distal to the MCA.^18, 19^ Moreover, only relative changes in compliance relating to changes in physiologic state (i.e., change in intracranial pressure^19^ or occlusion of the internal carotid artery^18^) are then reported, which confounds comparison with measurements of aBV changes over the cardiac cycle in distinct cerebral arteries as has been performed here.

Another cerebral compliance measure derived from TCD data is the augmentation index, which is calculated as the ratio of the difference between velocities at the return of the pulse wave reflected by the distal arterial bed and at diastole, and the differences between systolic and diastolic velocities in the flow profile of the insonated vessels (MCA and PCA in the experiment performed by Flück *et al*).^20^ Although detection of the pulse wave reflected by the more distal vascular bed is in part determined by the stiffness of the arterial wall at the location of insonation, because pulse wave velocity is linked to arterial stiffness, it is also highly influenced by the vessel branching and vessel wall properties of the distal arterial bed.^13, 20, 32^ In contrast to the results presented here, Flück *et al* report a higher augmentation index for the PCA (80.5%) than for the MCA (76.1%) in young individuals, suggesting that the PCA is less compliant. However, the dependency of the cerebral augmentation index on both the distal arterial bed and the stiffness of the arterial wall at the location of insonation inhibits the separate estimation of these parameters and again illustrates that our method gives complementary information to the TCD estimates of arterial stiffness.

Current models of pressure–flow relationships in the cerebral vasculature are in general validated with TCD measurements of blood flow velocity profiles through accessible cerebral arteries,^9, 10, 36^ and therefore also stress that the compliance parameters included in these models reflect the capacitive behavior of the distal arterial bed instead of local arterial wall properties.^9^ Furthermore, values used for AC vary in order of magnitude between different models. For instance, Alastruey *et al*^11^ use one-dimensional pressure–flow models of compliant arteries to model the circle of Willis and use values ranging from 8.3 *μ*L/mm Hg, for the arterial bed distal to the PCA, to 15.5 *μ*L/mm Hg for the arterial bed distal to the MCA. In contrast, Reymond *et al*^10^ also use one-dimensional models of pressure and flow to model the circle of Willis and use compliance values ranging from 0.028 to 0.058 *μ*L/mm Hg for the arterial beds supplied by the MCA and PCA, respectively. Although both studies use similar assumptions to calculate the compliances, including that the total systemic compliance is distributed over all compliances in the full model and that the local geometry of the arteries is taken into account, differences arise owing to the literature-based values each study uses for the cerebral geometry and total systemic compliance.^10, 11^ As an example, when combining our results of normalized AC with the volumes for arterial segments derived from the data used by Reymond *et al*,^10^ we would come to 0.15, 0.089, and 0.03 *μ*L/mm Hg for MCA M1, ACA A1, and PCA P1, respectively, values that fall within the range of compliances used in the modeling literature.^11^

Interestingly, a recent study performed by Yan *et al*^37^ also uses ASL to assess AC and reports changes in aBV in large cerebral arteries of ~46% over the cardiac cycle, while the AC values reported here correspond to a change in aBV over the cardiac cycle of ~23%, 28%, and 58% in the ACA, MCA, and PCA, respectively. Although Yan *et al*^37^ used a different ASL method to assess aBV, combining their results with the current AC values for local flow territories suggests that ASL-based methods for detecting changes in aBV related to the cardiac cycle are consistent.

In short, ASL-derived measurements of arterial stiffness are difficult to compare directly with compliance measurements of the cerebral vasculature derived from different methodologies, such as TCD. Our method of local AC measurement has the potential of giving complementary information to TCD-based estimates of compliance. Reports on *in vivo* local AC are scarce in the literature, but comparison to values derived from previous experiments seems to suggest the reported AC values found here are plausible. Future developments include assessing repeatability of our novel cerebral AC measurement.

### Methodological Considerations

In the current experiment no model of tissue perfusion is included, even though PASL is conventionally used to assess this physiologic parameter. The main reason for not including this compartment in the model is the fact that the TIs used here are below 1 second, which means that the majority of the labeled blood has not reached the capillary bed of the brain yet and therefore little perfusion of the label has taken place. However, at TI=850 ms some perfusion might have still taken place, since transit delays from the blood to the capillary bed of more proximal brain regions have been reported to be <900 ms.^27, 38^ Our simulation study described in the Introduction section aimed to address this and showed that a short arrival time of the label to the capillary bed (of 700 ms), would result in a maximum overestimation of aBV of 5%, when the underlying aBV is >1%_v_. As an example, the resulting aBV_Dia_ for the LPCA here (group average aBV_Dia_=1.68%_v_ with Δ*t*_Dia_=445 ms) indicates that this is an overestimation of 1.2%, meaning that the true aBV_Dia_ would actually be 1.65%_v_. This level of overestimation we deemed acceptable in this study.

It is important to note that, although overestimation of aBV is marginal, it is dependent on the true aBV and decreases with increasing aBV (as illustrated in Figure 2). This means that the estimates for aBV_Dia_ are more biased than the estimates for aBV_Sys_ and, because compliance depends on aBV_Sys_–aBV_Dia,_ the AC might therefore be underestimated. However, as illustrated by the example of the LPCA as stated above, the fact that the ROIs used here only contain voxels with a sufficiently large aBV (>1.5%_v_) ensures that not including later TIs has limited effect on the AC measurement.

One potential systematic error in this study could arise from using brachial BPs as a surrogate for intracranial BPs. Pulse pressure in the common carotid artery is lower than in the brachial artery, because the peripheral brachial artery is located closer to reflection sites of the pressure wave through the vascular tree.^13^ Use of the brachial pulse pressure could, therefore, result in an underestimate of cerebral AC. Distal to the carotid the pulse pressure decreases again until it is (almost) zero in the capillary bed to facilitate exchange of oxygen and nutrients in the cerebral tissue.^39^ The cerebral arteries of interest here lie in between the common carotid and the capillary bed, which means that an invasive measurement would be necessary to obtain local pulse pressures.

To obtain perfusion-weighted ΔM images within the ASL technique it is required to subtract tag from control images. The retrospective synchronization of image acquisition to the cardiac cycle used here can result in an unbalanced number of tag and control images used to calculate the ΔM images. When the number of tag or control images used to calculate the ΔM of a particular cardiac phase becomes too small, there is a potential to lead to biased measurements of aBV_Sys_ or aBV_Dia_. In particular when motion is present within the time series. However, because of the use of a variable repetition time in image acquisition and the constant heart rate of healthy participants in rest of the current data set, it does not reveal any bias toward the number of images used to calculate systolic and diastolic aBV maps.

Lastly, it should be noted that cerebral aBV can be measured with different MRI methods as presented here, some of which could be synchronized to the cardiac cycle and therefore have the potential to assess cerebral AC as well. For instance, Yan *et al*^37^ have already combined ASL with a balanced steady-state–free procession readout to assess AC within large arteries and smaller arterioles. One drawback of Yan's method is that it is a single-slice method, making it less time efficient as the multislice method we propose here for investigation of AC throughout the brain. Vascular space occupancy with dynamic subtraction is another method that can quantify aBV, by assessing differences between an image in which blood signal is nulled and an image without nulling of blood signal.^40^ However, vascular space occupancy is currently optimized to assess precapillary aBV on a single-slice basis and, because the time of imaging depends on the repetition time of the pulse sequence, would require adaption to assess aBV of larger arteries at different times within the cardiac cycle.

The current study has shown the feasibility of measuring local AC of cerebral arteries with short TI PASL and has resulted in plausible estimates of local AC. Compared with TCD, measurement of AC with PASL has the benefit of measuring AC based on local arterial wall properties instead of assessing compliance of the arterial bed distal to the location of measurement and therefore has the potential to give complimentary information about the health of cerebral arteries.

## Figures and Tables

**Table 1:** Summary of participant data (three M and two F)

*Subject*	*Age (years)*	*Sex (M/F)*	*BP (sys/dia*–*PP, mm Hg)*	*Heart rate (bpm)*	*Slice just above circle of Willis (Z number)*
I	28	M	122/56–66	59.8	4
II	24	M	119/62–57	50.6	4
III	25	F	108/66–42	71.7	3
IV	25	F	110/64–46	69.5	3
V	25	M	119/66–53	75.2	4

BP, blood pressure; bpm, beats per minute (averaged over seven acquisitions); dia, diastole; F, females; M, males; PP, pulse pressure; sys, systole.

Mean and s.d. are shown.
