# Supplementary material for: Noninvasive assessment of arterial compliance of human cerebral arteries with short inversion time arterial spin labeling
Source: J Cereb Blood Flow Metab. 2014 Dec 17;35(3):461–8. doi: 10.1038/jcbfm.2014.219 (PMC4348387; doi:10.1038/jcbfm.2014.219)
Supplement: Supplementary Figure 1 [file jcbfm2014219x1.pdf]

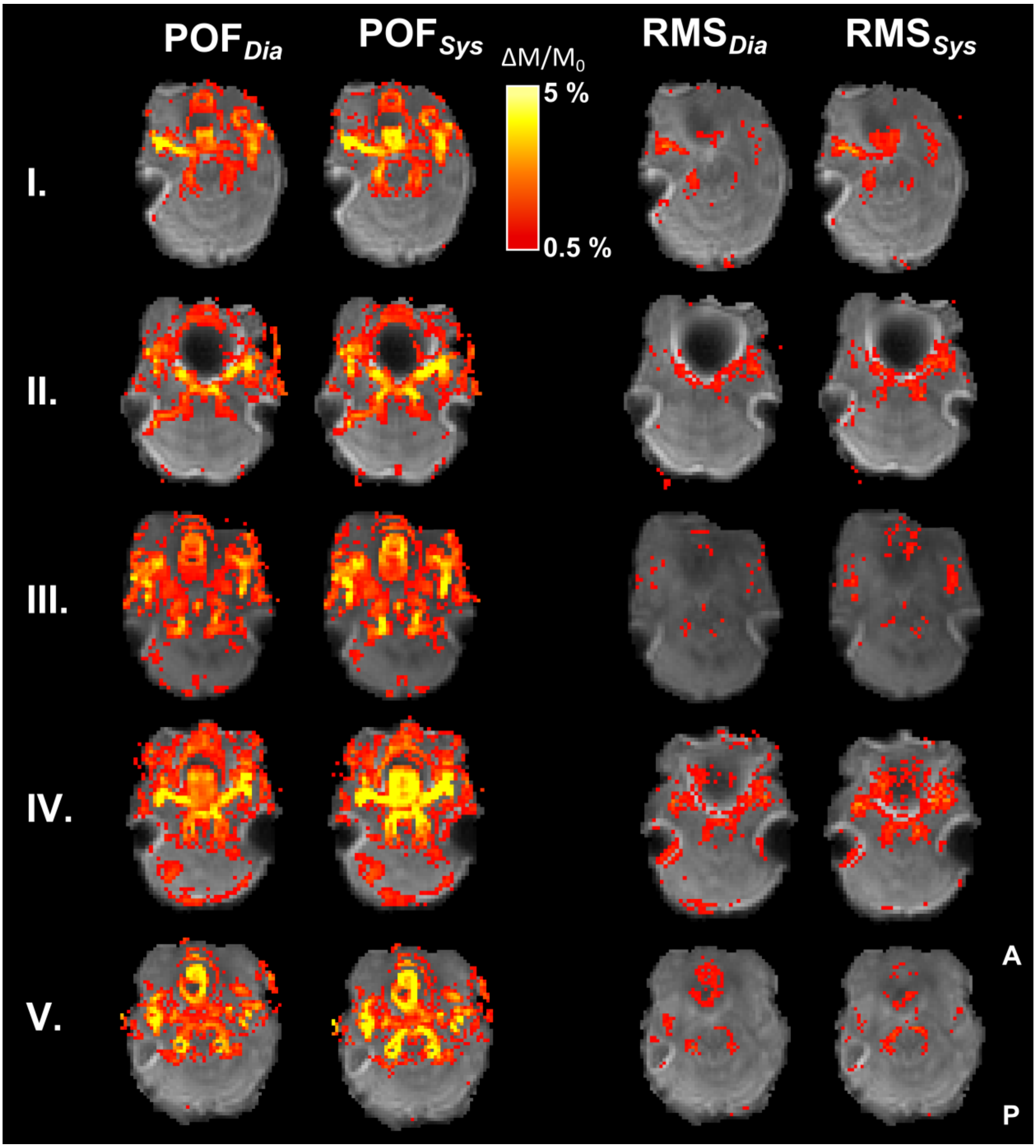

Supplementary Figure 1. Maps of the peak intensity of the fitted model (POF) and of the root mean square error (RMS) of the model fit in diastole (POF_{Dia} and RMS_{Dia}) and systole (POF_{Sys} and RMS_{Sys}) for all five subjects (I-V). The RMS is calculated according to:

$$RMS = \sqrt{\frac{\Sigma(\hat{y}(t_{acq})-y(t_{acq})^2}{n}},$$

where $\hat{y}$ is the model that fits arterial blood volume (aBV), arrival time (Δt), and dispersion (σ), t_{acq} is the time of slice acquisition (taking the slice delay of 29 ms into account), $y(t_{acq})$ is the average difference signal acquired at t_{acq} (i.e. the average $\Delta M/M_{0,a}$ signal at t_{acq}), and n is the number of time points (n = 7). Note that RMS is proportional to the amount of arterial signal in the voxel, which means that RMS in the arteries is higher than in the brain tissue. It can be seen here that the RMS are in the order of 0.1-1% of the equilibrium magnetisation of blood $M_{0,a}$, while the peak arterial signal is in the order of 5% of $M_{0,a}$ (maps of POF on the left). This means that the residuals of the model fit are < 20 % of the acquired signal.
