# Supplementary material for: Noninvasive assessment of arterial compliance of human cerebral arteries with short inversion time arterial spin labeling
Source: J Cereb Blood Flow Metab. 2014 Dec 17;35(3):461–8. doi: 10.1038/jcbfm.2014.219 (PMC4348387; doi:10.1038/jcbfm.2014.219)
Supplement: Supplementary Figure 2 [file jcbfm2014219x2.pdf]

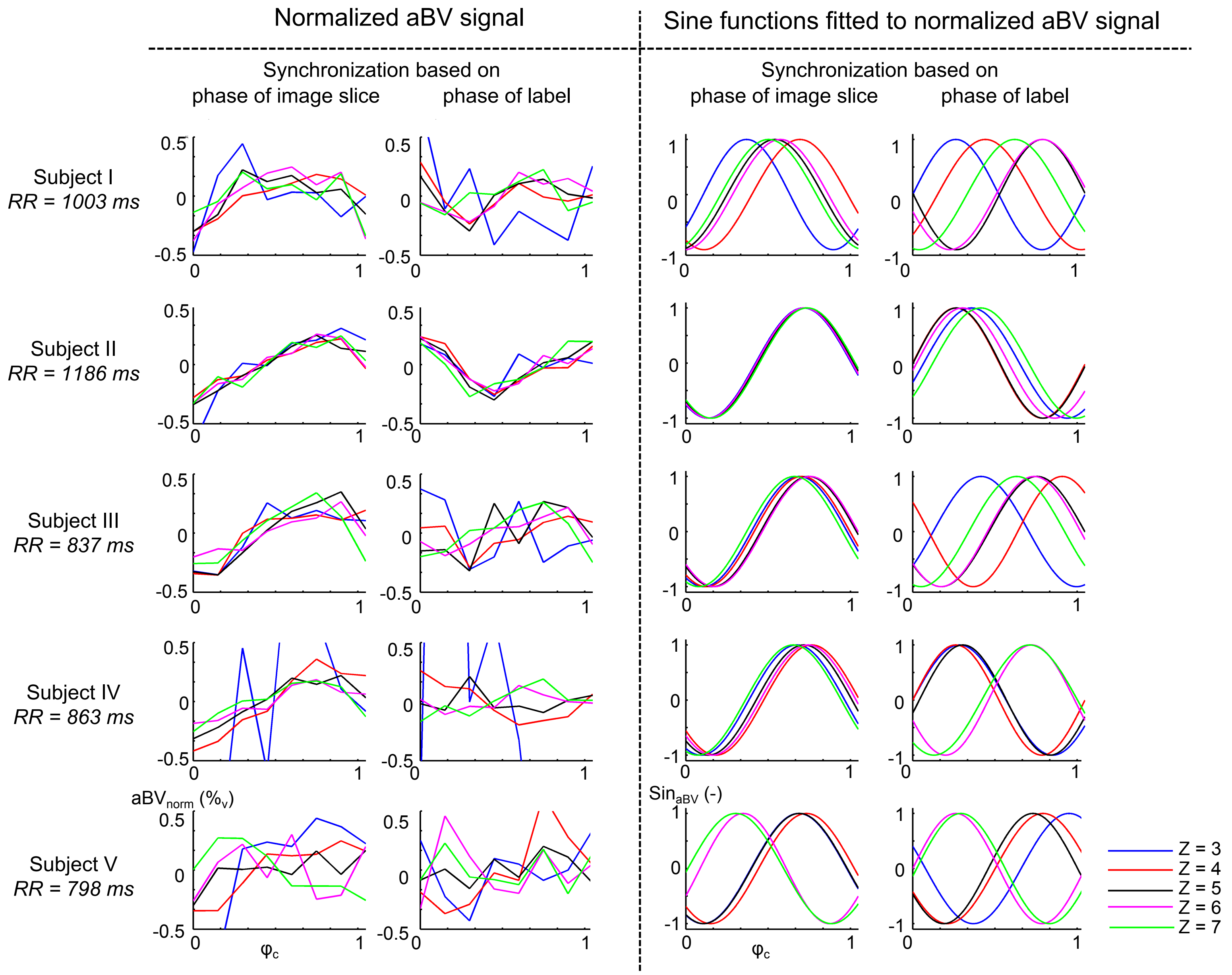

Supplementary Figure 2. To illustrate the dependency of arterial blood volume (aBV in volume percentage of the voxel: $\%_v$) on the cardiac phase (ϕ_c) of the *imaging* volume here results are shown for aBV in the flow territory of the left middle cerebral artery (LMCA) for 5 consecutive slices (coloured lines, Z = 3-7). Each row contains data from one subject. The two columns on the left show aBV_{Norm} (aBV - the average aBV over the cardiac cycle). The two columns on the right show sine waves fitted to the data on the left to highlight the difference between synchronisation based on cardiac phase of the *image* and *label* (amplitudes of the sine waves are set to 1). The cardiac phase for which aBV is maximum is similar for al 5 consecutive slices when the imaging data is retrospectively synchronized according to the phase of the *imaging* slice (column 1 and 3), because all slices are acquired during the same cardiac phase. When the data is retrospectively synchronized according to the phase of the *label* the cardiac phase for which aBV is maximum depends on slice location. If images are synchronized based on when the *label* has occured, there will be differences in cardiac phase of consecutive slices becasue a 2D acquisition scheme is used with a slice delay of 29 ms. In particular when the slice delay is relatively long compared to the duration of a single cardiac cycle (RR interval), the dependency of aBV on the cardiac phase of the imaging slice becomes more apparent (compare subject III with subject II). In subject III, with a relatively short RR interval, this effect is illustrated by the dispersion of cardiac phase in data synchronized with the cardiac phase of the *label* (column 4) compared to the data synchronized based on the cardiac phase of the *image* (column 3).
